# Pilot Study: Step Width Estimation with Body-Worn Magnetoelectric Sensors

**DOI:** 10.3390/s25113390

**Published:** 2025-05-28

**Authors:** Johannes Hoffmann, Erik Engelhardt, Moritz Boueke, Julius Welzel, Clint Hansen, Walter Maetzler, Gerhard Schmidt

**Affiliations:** 1 Department of Electrical and Information Engineering, Kiel University, 24143 Kiel , Germany; jph@tf.uni-kiel.de (J.H.); eren@tf.uni-kiel.de (E.E.); mobo@tf.uni-kiel.de (M.B.); 2 Department of Neurology, University Hospital Schleswig-Holstein Campus Kiel and Kiel University, 24105 Kiel, Germany; j.welzel@neurologie.uni-kiel.de (J.W.); c.hansen@neurologie.uni-kiel.de (C.H.); w.maetzler@neurologie.uni-kiel.de (W.M.)

**Keywords:** gait analysis, gait variability, magnetic motion tracking, magnetoelectric sensor, technical validation

## Abstract

Step width is an important clinical motor marker for gait stability assessment. While laboratory-based systems can measure it with high accuracy, wearable solutions based on inertial measurement units do not directly provide spatial information such as distances. Therefore, we propose a magnetic estimation approach based on a pair of shank-worn magnetoelectric (ME) sensors. In this pilot study, we estimated the step width of eight healthy participants during treadmill walking and compared it to an optical motion capture (OMC) reference. In a direct comparison with OMC markers attached to the magnetic system, we achieved a high estimation accuracy in terms of the mean absolute error (MAE) for step width (≤1 cm) and step width variability (<0.1 cm). In a more general comparison with heel-mounted markers during the swing phase, the standard deviation of the error (<0.5 cm, measure for precision), the step width variability estimation MAE (<0.2 cm) and the Spearman correlation (>0.88) of individual feet were still encouraging, but the accuracy was negatively affected by a constant proxy bias (3.7 and 4.6 cm) due to the different anatomical reference points used in each method. The high accuracy of the system in the first case and the high precision in the second case underline the potential of magnetic motion tracking for gait stability assessment in wearable movement analysis.

## 1. Introduction

Spatiotemporal gait parameters are a key component in clinical movement analysis for mobility-limiting conditions such as Parkinson’s disease (PD) [1,2]. The aspect of mediolateral (side-to-side) movements is crucial for gait stability assessment and fall prevention [3,4]. Step width can be conceptualized as a surrogate marker for various aspects of mediolateral movement. In certain pathological conditions, such as ataxia, an increase in step width (by 6 cm [5]) is observed due to impaired control over the body’s center of mass [6]. In other conditions, such as PD, a reduction in step width is observed [7]. Furthermore, the variability of step width (standard deviation) is regarded as a motor marker associated with postural instability [8,9].

Step width is one parameter in spatiotemporal gait analysis [10], which is commonly performed in the clinical motor laboratory using three different types of sensor systems.

Optical motion capture (OMC) is based on infrared camera systems that provide trajectories of body-worn reflective markers in the 3D space [11], from which step width can be derived based on step times (temporal component) and distances (spatial component). Electronic walkways consist of pressure-sensitive plates to similarly detect and localize gait events [12]. Full-body motion suits, e.g., based on 16 body-worn inertial measurement units (IMUs), are also used in clinical gait analysis [13,14].

Application in the patient’s usual environment (home assessment) is critical for comprehensive movement assessment [15] but usually not applicable to these systems designed for laboratory use. For the subsequent comparison of different approaches, we focus on the error between the wearable system and the reference. We consider the mean absolute error (MAE) as a measure of accuracy and the standard deviation (SD) of the (signed) error as a measure of precision (consistency).

Although there are efforts to apply IMU-based multi-sensor systems in real-world conditions [16], portable setups consisting of only a few IMUs are more commonly used, e.g., for step length and step time estimation [17,18]. However, the relative nature of step width (relative distance between both feet) presents a challenge to IMU-based measurement systems [19]. There are two categories of methods to overcome this limitation: Software-based approaches use complex models based on limited IMU setups. Deep learning methods can model the complex relation between IMU data and step width [20,21]. Wang et al. achieved a step width estimation accuracy of 3.3 cm (mean absolute error, MAE) in individuals with Spinocerebellar ataxia using a data-augmentation scheme that relies on training data from healthy and pathological individuals [22].

Hardware-based approaches augment the IMU results with additional reference sensors that can provide spatial estimates directly (sensor fusion). Anderson et al. used a network of four foot-mounted ultra wideband (UWB) transceivers to estimate step width with an accuracy of 3.3 cm compared to an instrumented walkway [23], and another approach achieved 0.76 cm for foot clearance [24]. Foot-mounted ultrasound transducers can also be used in combination with IMUs for step width estimation, as demonstrated by Weenk et al. with 1.2 cm MAE [25]. Infrared-based tracking can provide <1 cm accuracy compared to an OMC system [26] or even better results in a scenario in which leg clearance acts as a proxy for step width [27]. Chheng et al. applied magnetic tracking with a Hall-effect sensor for abnormal gait detection with leg clearance as a stand-in for step width [28].

Generally, magnetic tracking can be applied with passive (permanent magnet [29]) or active (coil [30], permanent magnet cantilever [31]) magnetic field sources. The active method has been previously applied in general-purpose motion tracking of the upper extremities as it allows for the separation of channels and longer ranges [32]. Compared to infrared and ultrasonic approaches, magnetic tracking does not require line-of-sight conditions [33]. As the main advantage, this allows magnetic trackers to be worn discreetly under clothing and remain unaffected by real-world conditions, such as rain, fog or dust.

In principle, magnetic tracking is not limited to a specific type of sensor. However, in our approach, we operate AC magnetic fields close to the sensors’ resonance frequencies of approximately 7.7 kHz. This avoids parasitic low frequency fields and power line harmonics, while allowing frequency multiplexing. The applied 3 mm × 1 mm sensor elements are early-stage demonstrators produced with clean-room technology as part of the Collaborative Research Center 1261 on Biomagnetic Sensing [34,35]. This fabrication process has potential for the wafer-level integration with micro-electromechanical system (MEMS) sensors (e.g., accelerometers) for sensor fusion. The sensor type is part of a larger family of emerging magnetoelectric sensors [36,37,38,39].

In this paper, we bridge the gap between our previously presented general-purpose magnetic motion tracking system [40] and the estimation of step width as a clinically relevant motor marker. We conceptualized and implemented a geometric approach to derive step width from the magnetic distance estimates. We applied the adapted system in a pilot study of treadmill walking with eight healthy participants. Using OMC markers attached to the participant’s heels (anatomical markers) and to the magnetic tracking devices (technical markers), we validated the spatial and temporal performance of our approach with the goal of gait stability assessment in real-world conditions.

## 2. Materials and Methods

Step width is broadly defined as the mediolateral distance between both feet, but the applied interpretation varies between the previously discussed hardware-based approaches. It has to be considered in the context of the human gait cycle [41], which consists of alternating stance and swing phases for both feet. The stance phase is initiated when a foot hits the ground (initial contact). It remains in contact with the ground through the mid- and terminal-stance. Then, the swing phase is initiated: the foot is lifted and passes by the other leg (mid-swing), and with the next initial contact, the cycle starts again. For the definition of step width, multiple aspects must be taken into account:

(1) Timing: To which phase (or phases) of the gait cycle does the step width refer?
(2) Distance: What distance (in which coordinate frame) is considered?
(3) Placement: What point on the feet (or even legs) is used as the reference point?

An informed decision on these aspects must incorporate details of the applied sensor system.

### 2.1. Measurement System

We previously introduced a modular motion tracking system based on ME sensors [40]. The system consists of up to four sensor nodes (3D ME sensor arrays) and up to two actuator nodes (triaxial square coils). Each node is a 3D-printed cube with an edge length of 35 mm, which is attached to carrier PCBs on two sides. These boards handle the wiring and feature mounting holes with Velcro straps and optical markers. The Experimental Setup subsection contains placement details and pictures. Each actuator channel is typically driven by a sinusoidal signal of 1.2 V and 480 mA (both RMS). These are operated in a frequency-division multiple access scheme with 50 Hz spacing near 7.7 kHz.

**Figure 1:**
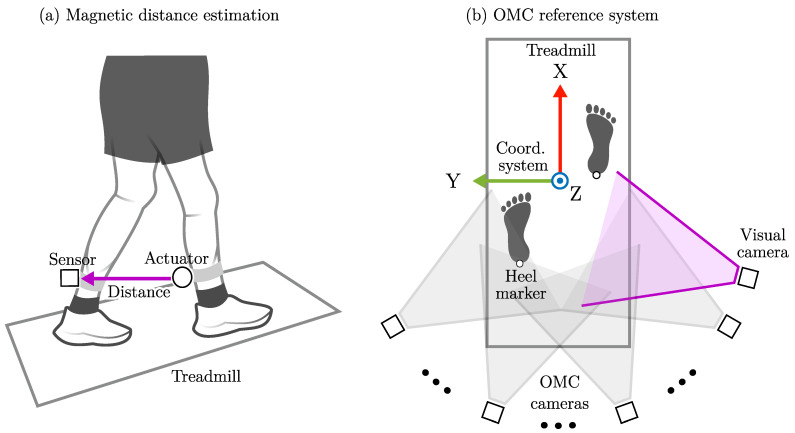
Magnetic distance estimation system and optical reference system. The magnetic estimate corresponds to the actuator–sensor distance during treadmill walking (**a**). The OMC reference system tracks the reflective markers inside the measurement volume with the coordinate system aligned with the treadmill (**b**).

In the previous study, we were able to measure distances between multiple nodes attached to the lower extremities and toward a fixed infrastructure. The accuracy was below 1 cm during treadmill walking with the range varying for the individual actuator–sensor pairs between 40 cm and 60 cm. The goal of this study is to apply an adapted version of this motion tracking system in the context of spatiotemporal gait analysis. With this in mind, we first had to define how the abstract distance estimates (Figure 1a) could be transformed into a meaningful clinical motor marker. Therefore, we analyzed existing methods and derived a concept for the magnetic approach.

For reference purposes, all magnetic recordings were performed in an eight-camera OMC measurement volume (Miqus M3, Qualisys AB, Gothenburg, Sweden) at 100 Hz. The trajectories were aligned with the treadmill coordinate system. The subsequent visualizations of step width definitions were based on heel marker trajectories during a full gait cycle at 0.5 m/s (Figure 1b). The treadmill movement was subtracted from the corresponding trajectory component.

### 2.2. Step Width: Initial Contact Method

**Figure 2:**
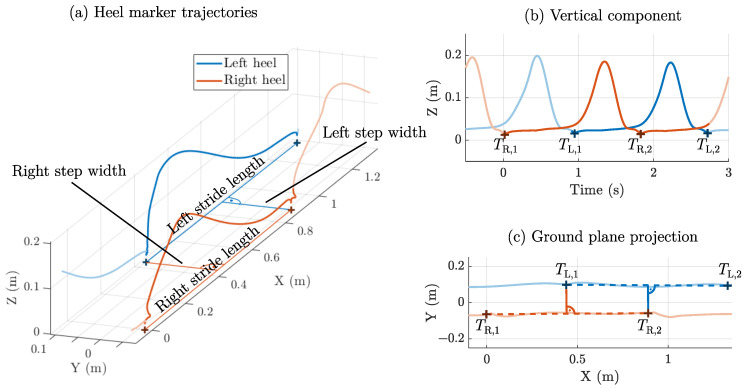
Step width assessment during initial contact. The heel marker trajectories are depicted with stride length and width (**a**). Two subsequent initial contacts for each foot (right: $T_{R},1/2$, left: $T_{L},1/2$) were detected from the minima in the vertical trajectory component (**b**). The resulting projection on the ground shows the assumption of linear progression compared to the mediolateral distance (**c**).

As a starting point, we refer to a general definition of step width for both linear (straight line) and non-linear walking [42]. The same point on both feet must be considered for a meaningful distance estimate. OMC-based assessment typically uses markers at the center of the heel (calcaneus). The stride length for each side is defined as the distance between the positions of two subsequent initial contacts of the same foot. Step width is defined as the perpendicular distance to the intermediate initial contact of the other foot. For linear walking, the line of progression is often assumed to point forward, leading to a simplified calculation. Figure 2 highlights the relation of the trajectories to stride length and step width. This definition assumes a linear progression of the same foot between two initial contact phases, which can deviate from the actual mediolateral movement during the swing phase (Figure 2c).

In summary, this method considers the absolute position during initial contact on three separate occasions. This method is tailored for absolute localization systems such as instrumented walkways or OMC but not easily applicable to a system based on relative distance estimation between two body-worn tracking devices. The tracking distance is assumed to be the vector norm of the difference of both marker trajectories.

### 2.3. Step Width: Mid-Swing Method

**Figure 3:**
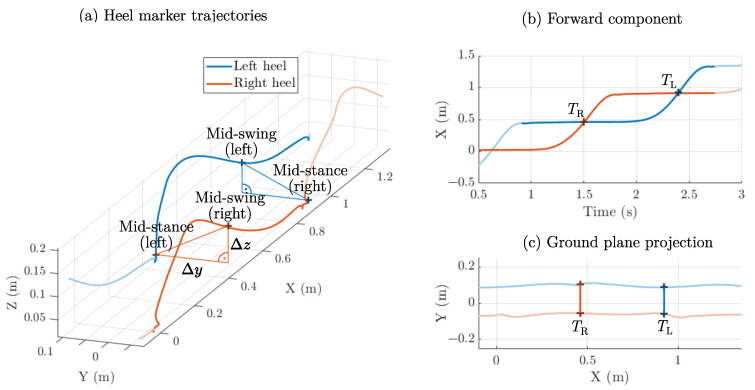
Step width assessment during mid-swing. The heel marker trajectories (**a**) are depicted with the mediolateral Δy and vertical distance Δz (during a right step). The corresponding events were detected from the intersections at times $T_{R}$ (right step) and $T_{L}$ (left step) in the forward component (**b**). The resulting projection onto the ground plane is depicted in (**c**).

For a distance estimation system, it is more appropriate to assess the mediolateral (side-to-side) distance at one specific time point. Accordingly, most hardware-based approaches consider the moment in which one foot is in mid-stance and the other one passes by it (mid-swing). In Figure 3, and the heel marker trajectories are displayed with the specific points emphasized. This measurement method corresponds to a sampling of the mediolateral distance at one specific point in the ground plane projection of the trajectories (Figure 3c).

The disadvantage of tracking in the swing phase is that a pure distance estimate between both feet or lower shanks contains not only a mediolateral component but also a vertical component. First, the minimum in the tracking distance (both components) must align temporally. Secondly, the vertical component must be compensated for. The hardware approaches discussed previously work around this by either using a reflector (transmitter and receiver on one leg and receiver on the other leg) [26] or placing the sensor on the thigh, which reduces vertical movement compared to the feet [28]. For the optical signals, the trajectory components can easily be separated, so this method is well suited as a reference in the validation of the magnetic approach.

### 2.4. Step Width: Shank Clearance Method

**Figure 4:**
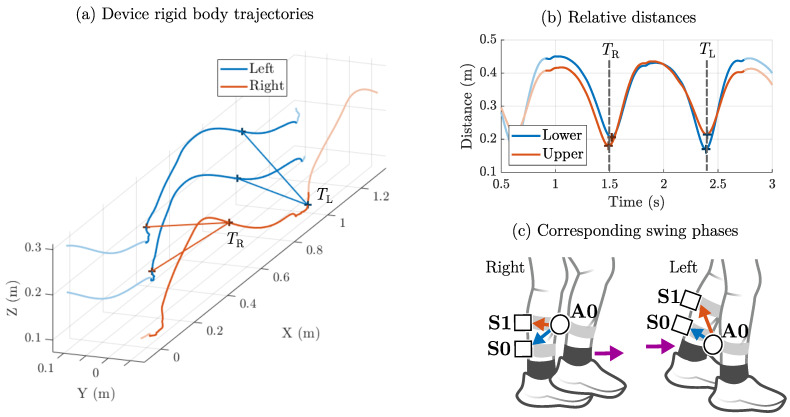
Step width assessment based on shank clearance minima. The device marker trajectories are depicted with lower and upper tracking distances during right ($T_{R}$) and left ($T_{L}$) swing (**a**). The relative distances show corresponding minima (**b**). The detected events qualitatively correspond to the displayed swing phases with two shank-worn sensors (S0 and S1) and a shank-worn actuator (A0) (**c**).

Based on the previous method, we conceptualized a magnetic tracking approach that can handle vertical movements. We decided on a subset of the modular magnetic motion tracking system based on two sensor nodes (S0 and S1) and one actuator node (A0) (cf. Figure 4). Shoe- or foot-mounted magnetic devices seemed impractical for the study (due to shoe size and shock protection), so we chose a strap-based attachment to the shanks with two (rigidly connected) sensors on the left shank and one actuator on the right shank. Instead of a direct assessment of step width at the feet, leg clearance is used as a proxy, similar to [27]. Figure 4 illustrates how this method works in principle. Here, we consider the trajectories of the magnetic devices, extracted from clusters of reflective markers (rigid bodies, cf. Figure 4a). Minima in the relative distances for lower and upper sensor are used for event detection (Figure 4b). When the right foot moves forward in the swing phase ($T_{R}$), the distance to the upper sensor is closer than to the lower sensor (Figure 4c). The same consideration applies for the left swing phase ($T_{L}$). In the resulting sequence of alternating minima, right and left steps can be differentiated.

**Figure 5:**
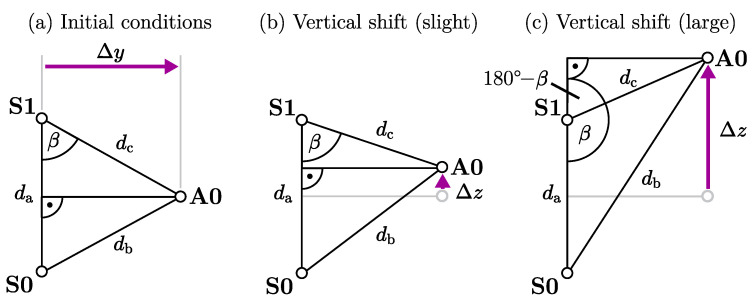
Geometric approach for the magnetic step width estimation. The desired horizontal distance is the height Δy of the triangle perpendicular to $d_{a}$. In the initial state (**a**), there is no vertical shift Δz. For a slight upward shift (**b**), angle β remains below 90^∘^. For a larger increase (**c**), β is above 90^∘^.

Based on these considerations, the geometric relations between the tracking distances $d_{b}$ and $d_{c}$ can be derived (Figure 5). The inter-sensor distance $d_{a}$ is constant and known a priori. The sides $d_{b}$ and $d_{c}$ form a triangle with the known side $d_{a}$ (Figure 5a). We look for the distance Δy, which is the height of the triangle perpendicular to $d_{a}$. Based on the law of cosines, the angle β is defined as:

(1) $$\beta =\arccos\left(\frac{d_{a}^{2}+d_{c}^{2}-d_{b}^{2}}{2d_{a}d_{c}}\right).$$

For 0^∘^≤β≤ 90^∘^, the horizontal distance Δy is available via a sine relation with the angle β and $d_{c}$. This corresponds to a downward shift or a slight upward shift (Figure 5b). For 90^∘^ ≤β≤ 180^∘^ (Figure 5c), the adjacent angle (180^∘^− β) is considered instead with ($\sin\left(180^{\circ }-\beta \right)=\sin\left(\beta \right)$). Thus, the resulting equation for Δy in both cases is as follows:

(2) $$\Delta y=d_{c}\sin\left(\beta \right).$$

We define Δz as the vertical shift of A0 relative to the center between S0 and S1 (Figure 5b), with a positive sign for upward shifts and a negative sign for downward shifts β:

(3) $$\Delta z=\frac{d_{a}}{2}-d_{c}\cos\left(\beta \right).$$

This equation system can now be applied to separate the mediolateral distance from the vertical distance by combining both distance estimates (${\hat{d}}_{b}$ and ${\hat{d}}_{c}$) from the magnetoelectric motion tracking system. The resulting Δy is what we consider as step width in the shank clearance method.

### 2.5. Signal Conditioning and Feature Extraction

Apart from the spatial considerations, the relevant time events must be detected to estimate step width as described. This has implications for the magnetic motion tracking system, which is driven by a digital signal processing framework including a sensor-level and system-level calibration. Our previous publication [40] suggests that an appropriate calibration training set should contain data in the relevant spatial volume for the application. Accordingly, we applied a new calibration set targeting movements ≤20 cm away from the sensor for various orientations.

The magnetoelectric sensors measure the magnetic signal transmitted by the coils, which corresponds to the desired signal component in this scenario. Generally, the magnetic field strength of the coil (dipole approx [43]) decays with the distance *r* with 1/$r^{3}$. The bandwidth of the motion tracking system is 25 Hz, so the provided distance estimations contain the fundamental frequencies of locomotion and the corresponding harmonics. In addition, the sensor picks up noise from various sources, representing the undesired signal components. High-frequency mechanical oscillation occurs during walking in the lower limbs [44]. As ME cantilever sensors are sensitive to vibration (especially shock) [45], the effect on the signal-to-noise ratio (SNR) must be considered. We assume that the performance of the magnetic system varies with the phase of the gait cycle depending on distance and noise:

(1) Mid-swing: The distance between both shanks is rather short (≈20 cm), and the foot in swing phase has not yet hit the ground (high signal, low noise).
(2) Initial contact: The distance between both shanks reaches its maximum (≈40 cm), and the foot hits the ground (low signal, high noise).

**Figure 6:**
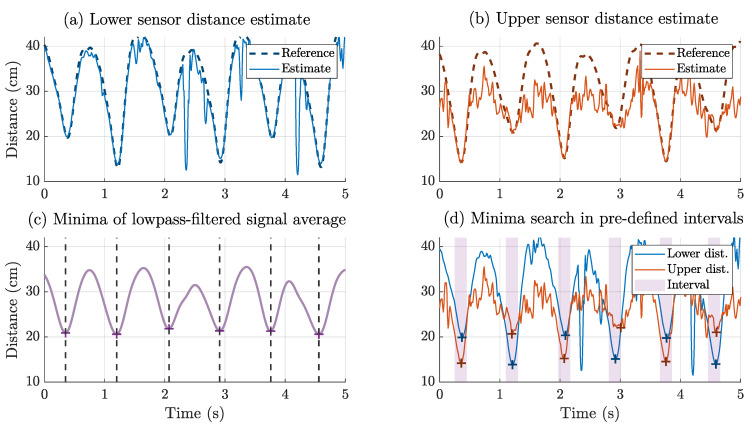
Exemplary distance estimation results to highlight the feature extraction process. Lower (**a**) and upper (**b**) sensor distance estimates with optical reference. Data from Participant 2, starting at 1.35 s. The low-pass-filtered average signal (**c**) of both distances was used to detect minima (‘+’, dashed line). Each minimum was used as the center of an interval (shade), in which the minima of the lower and upper distance signals (‘+’) were detected (**d**).

Consequently, there are parts of the sensor signal dominated by noise. Figure 6a,b compare the magnetic estimates to the optical reference for an exemplary result, in which the gait phase-dependent behavior is clearly visible. The undesired signal components must be suppressed for a reliable event detection and feature extraction. Therefore, we introduce a two-stage minima detection process that suppresses high-frequency and asymmetric signal components. As an auxiliary signal, we average both sensor signals sample by sample and apply a Butterworth low-pass filter (2 Hz, 2nd order, zero-phase):

(4) $${\hat{d}}_{\mathrm{lp}}\left(n\right)=\frac{1}{2}\left({\hat{d}}_{b}\left(n\right)+{\hat{d}}_{c}\left(n\right)\right)*h_{\mathrm{lp}}\left(n\right).$$

The resulting signal (Figure 6c) is expected to represent the fundamental frequency component during walking but neglect (both desired and undesired) components at higher frequencies. The swing phases are assumed to correspond to the local minima of the signal. Here, $k_{i}$ represents the index of the *i*-th minimum with a minimum spacing between the minima specified as 500 ms ($N_{0}$ samples):

(5) $${\hat{d}}_{\mathrm{lp}}\left(k_{i}\right)<{\hat{d}}_{\mathrm{lp}}\left(n\right)\;\mathrm{for}\;\mathrm{all}\;\;n\in \left[k_{i}-N_{0},\;k_{i}+N_{0}\right].$$

For each minimum identified in ${\hat{d}}_{\mathrm{lp}}$, we also expect a corresponding minimum in both ${\hat{d}}_{b}$ and ${\hat{d}}_{c}$ (Figure 6d). Each minimum $k_{b,i}$, $k_{c,i}$ is assumed to be within an interval of 100 ms ($N_{1}$ samples):

(6) $${\hat{d}}_{b}\left(k_{b,i}\right)\leq {\hat{d}}_{\mathrm{lp}}\left(n\right)\;\mathrm{for}\;\mathrm{all}\;\;k_{b,i},n\in \left[k_{i}-N_{1},\;k_{i}+N_{1}\right],$$

(7) $${\hat{d}}_{c}\left(k_{c,i}\right)\leq {\hat{d}}_{\mathrm{lp}}\left(n\right)\;\mathrm{for}\;\mathrm{all}\;\;k_{c,i},n\in \left[k_{i}-N_{1},\;k_{i}+N_{1}\right].$$

This minima search is performed on the unfiltered distance signals with the full 25 Hz bandwidth to include higher frequency components by harmonics. The resulting distance estimates at the selected indices are fed into the step width estimation to obtain the step width estimate $\hat{\Delta }y$.

### 2.6. Experimental Setup

**Figure 7:**
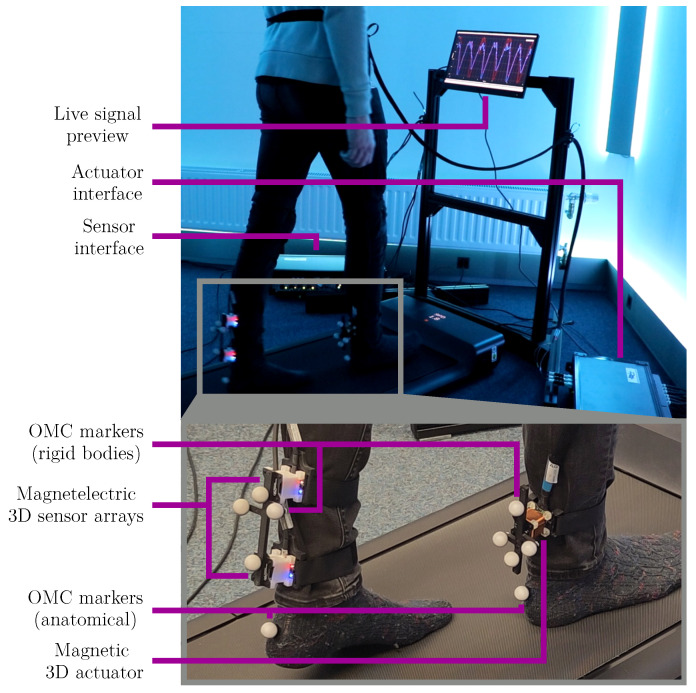
Experimental setup for magnetic step width estimation.

The experimental setup (Figure 7) consists of the treadmill (WalkingPad A1 Pro, KingSmith Co. Ltd., Beijing, China) with the sensor and actuator interfaces. A measuring stand in front of the treadmill was used for cable management purposes. The attached display was connected to a notebook running the applied real-time signal processing framework KiRAT [46], providing a live signal preview for technical purposes. During the gait trials, it was deactivated to avoid influencing the participants. The magnetic tracking devices were equipped with clusters of reflective markers for the OMC reference. Additional markers were placed on both heels for the mid-swing and initial contact reference methods.

The actuator and sensor nodes were attached to the shanks with Velcro straps. Both sensor nodes were connected by a solid, 5 mm thick plastic bar to ensure a constant inter-sensor distance of 10 cm. The weight of the combined sensor unit was 110 g. The magnetic devices were positioned with both feet in a natural position next to each other. The lower sensor node was placed on a vertical line approximately 5 cm above the left heel marker. The actuator node was placed on a vertical line above the right heel marker so that it was centered between the two sensors. Its weight was 85 g.

### 2.7. Error Metrics

We calculated several error metrics [47] to compare the magnetic estimates and the optical reference values for each participant. The mean and the standard deviation (SD) of the signed error were considered as measures for bias and spread of the error signal. The mean absolute error (MAE) and the root mean squared error (RMSE) were included as accuracy measures. The Spearman correlation coefficient (SCC) was added as an auxiliary metric for rank agreement. The mean absolute error of the step width variability (MAE-VAR) was computed as the absolute difference between the standard deviations of the optically and magnetically determined step widths. For the step width estimates, three subsets of steps were considered separately for each participant (left steps, right steps, and all steps). The metrics were averaged across participants for each subset. This was performed by standard averaging (bias, MAE, SCC, and MAE-VAR) or by root mean squared (SD and RMSE). The weighted average was used to account for the different number of steps for each participant.

## 3. Results

### 3.1. Dataset Overview

Table 1 summarizes the key facts of the pilot study. For each participant, 120 s of treadmill walking at 0.5 m/s were recorded. Surplus steps at the end were removed to ensure an equal number of left and right steps. Stimpson et al. indicate that an excessive (≥1 m/s) or insufficient (≤0.2 m/s) walking speed has large effects on step width variability [48]. Accordingly, we chose a moderate treadmill speed of 0.5 m/s. Table 1 shows step width and step width variability as calculated by the initial contact method, which is the most common approach. These values are only intended to provide an overview of the expected range of values.

### 3.2. Spatial Performance of the Magnetic Distance Estimation

The resulting spatial features were first compared to the reference distances obtained from the technical OMC markers on the sensors and the actuator. Table 2 focuses on performance across all participants by comparing the estimates of the lower and upper distance to the reference (not the derived step width).

### 3.3. Temporal Performance of the Gait Event Detection

Both the shank clearance approach and the mid-swing approach rely on accurate detection of the relevant timing events, i.e., when one foot is in mid-stance and the other foot is in mid-swing. For that purpose, we compared the magnetic detection times with the corresponding OMC results. Table 3 summarizes the results related to both the shank clearance and the mid-swing reference. As the sample rate was 100 Hz, 10 ms corresponds to one sample of shift. Overall, the detection accuracy in terms of the MAE is below two samples in each case. The initial contact method uses different time events, so no temporal validation with this method was applied.

### 3.4. Step Width Estimation Performance

**Figure 8:**
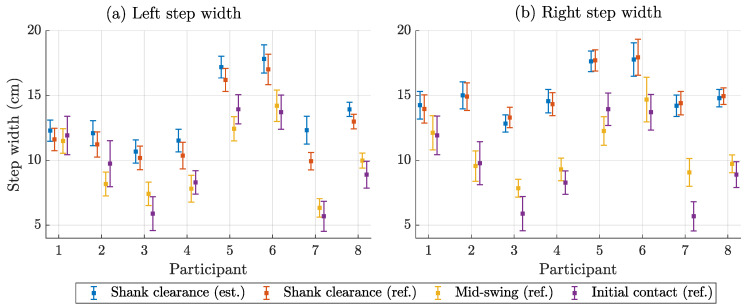
Distribution of step width by method and participant for left (**a**) and right (**b**) steps. Displayed as mean (square) with standard deviation (error bars).

**Figure 9:**
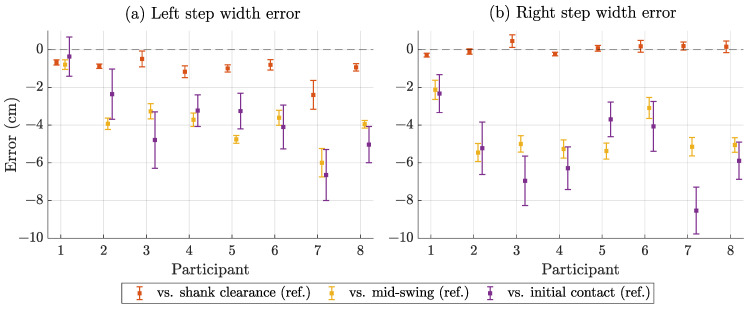
Step width estimation error compared to different reference methods by participant for left (**a**) and right (**b**) steps. Displayed as bias/mean error (square) with standard deviation of error (error bars). Dashed line at zero bias.

**Figure 10:**
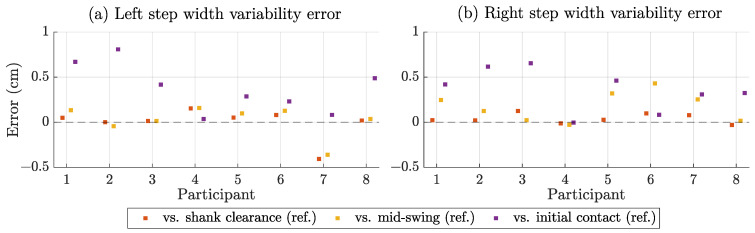
Step width variability estimation error compared to different reference methods by participant for left (**a**) and right (**b**) steps. Dashed line at zero bias.

To assess accuracy and precision, we compared the magnetic step width estimates with the three reference methods described above. We report the participant-specific results in the following plots. Figure 8 shows the descriptive statistics of the estimated step width in terms of mean value and standard deviation by participant and side. The mean step width ranged from 5 cm to 18 cm with large differences between participants and minor differences between left and right. Figure 9 compares the estimated step widths with the references in terms of bias (mean error) and standard deviation. The bias compared to shank clearance was by far the lowest, while mid-swing and initial contact were more similar. The SD for shank clearance and mid-swing was <1 cm, while it was 1–2 cm for initial contact. Figure 10 shows the errors when comparing the estimated step width variability with the different reference methods. The error for mid-swing was similar to shank clearance (few mm) for the left steps and slightly higher for the right steps. The error for initial contact was generally higher (mostly >3 mm).

Table 4 represents the averages of the participant-specific results shown in Figure 9 and Figure 10. It considers both sides separately and in combination.

## 4. Discussion

In this paper, we investigated a magnetoelectric distance estimation system to assess a clinical motor marker, i.e., step width. Most of the published software and hardware approaches achieve a distance estimation accuracy in the centimeter range [22,23,25], which is reasonable when considering the typical magnitudes of step width above 10 cm and the challenges due to sensor placement and calibration. The required accuracy of estimation may vary depending on the specific clinical condition of interest. We compared the presented ME system to three different reference methods (shank clearance, mid-swing, and initial contact) to obtain a comprehensive overview of the system’s capabilities and limitations. Because some of the results are affected by the different biases for left and right steps, we focus on the individual results for each.

In a first validation step, we compared the magnetic distance estimation directly to the distance readings obtained from the device-attached clusters of rigid bodies (shank clearance). With MAEs of ≤0.5 cm, the system maintained the previously described accuracy of below 1 cm for more general gait trials [40]. SCCs very close to 1 confirmed the high accordance with the optical results. This underlines the system’s capability to measure relative distances during walking at a high accuracy. Precision, as indicated by the standard deviation of the error (SD) of 0.3 cm, was similarly high.

Furthermore, we investigated the temporal accuracy of the system in detecting the minima of the distance signals, once again compared to the minima detected in the optical shank clearance results. Here, the accuracy showed an MAE near 10 ms corresponding to one sample. Even when comparing the minima to the intersections in the forward progression as obtained from the heel markers (mid-swing method), the MAE remained below two samples. This demonstrated that the system can detect the specified point in time (one foot in mid-stance and the other foot in mid-swing).

As another part of the validation, we compared the magnetic step width estimation with the three described reference methods. For the shank clearance method, the MAE was ≤1 cm for both sides. The performance was generally better for the right step width, which was probably caused by the the worse alignment of the minima for the left swing phase. While this set of values may be comparable to other participants examined with exactly this system, the comparability with metrics obtained with other systems is limited.

We also compared the magnetically estimated step width with the reference values obtained from the heel markers during mid-swing. In this scenario, the SCC for the individual legs was still close to 0.9. However, both ME and MAE had values close to 4 cm, which is clearly worse than in the previous scenario. The high mean value also negatively affects the metrics across all steps, which is worse than for the individual feet. However, the precision in terms of the SD was still below 0.5 cm, which is very similar to the first scenario. This indicates that the limited accuracy is caused by a constant overestimation, likely due to the use of the shank clearance as a proxy for step width, which might induce a proxy bias due to the position and orientation of the sensors relative to the heels. This applies similarly to any hardware approach that does not use tracking devices at an anatomical reference point (such as the heels) or adjusts for this deviation.

For the scenario in which step width was assessed during initial contact, the MAE itself was also in the range of 4–5 cm; however, the SD was also above 1 cm and the correlation for both sides was near 0.4. The comparison was mostly added for completeness as the assessment of step width during initial contact is fundamentally different than during swing phase.

We were also able to estimate step width variability, which is a common marker of gait stability [8]. For shank clearance, we achieved MAEs for the individual sides <1 mm, for mid-swing of 1–2 mm and for initial contact of ≤4 mm. This suggests a good agreement between the magnetic method and reference values obtained from shank clearance and mid-swing. Still, some caveats apply as the step width variability across participants was quite similar (1.26 ± 0.26 cm, cf. Table 1 and Figure 8), which was facilitated by the fact that the number of steps was relatively low (<150), and only healthy participants were included. The performance of step width variability estimation should be further investigated in a follow-up study with longer walking sequences of at least 400 consecutive steps [49] in both healthy and pathological participants.

Overall, the demonstrated performance was encouraging for further application of the system in a clinical context. However, some limitations regarding the experimental setup, the method and sensor type and the further development toward a fully wearable system remain. This pilot study has some constraints due to the protocol and setup that must be considered. It was conducted with only one walking speed and a small number of participants, which also limits the significance. This relatively low walking speed and anatomical differences in the participants could have contributed to the high inter-participant variability as it might be lower than the usual walking speed of the participants. The body-worn sensors were connected to the interfaces by cable, which did not allow for the application without a treadmill. The wiring and the size of the devices could also unintentionally influence the posture during walking. Also, more complex gait patterns, such as non-straight-line walking, could not be assessed.

Some additional limitations are linked to the sensing method and ME sensor type. Range is generally a limitation of magnetic tracking due to the decay of the magnetic field with the third power of the distance. As visible in Figure 6b, the system could track the distance between both shanks during mid-swing but not during initial contact. Continuous tracking throughout the whole gait cycle would be a prerequisite for continuous tracking of gait kinematics, which would also provide other motor markers such as step length. Further improvements in ME sensor design and integration (such as regarding acoustic noise compensation) would be beneficial to achieving this. The sensors were operated using electrically shielded housings and cables to minimize adverse effects by nearby electrical devices, such as the treadmill. However, we cannot completely rule out any influence. Nearby metal objects may also negatively affect the tracking performance [50].

For larger-scale experiments, a further integration of the sensor and actuator units into a battery-powered, wearable system would be required. This would also be beneficial for walking at treadmill speeds ≥ 0.5 m/s. While additional experiments indicate that higher walking speeds are possible with the current system, the cables pose some limitation on the participant and the sensor performance. There are no fundamental arguments against a wearable magnetic motion tracking system, but the power consumption of the coils (≈1.5 W) poses some limitations. Integration and miniaturization would also require some additional hardware engineering efforts, but this is beyond the scope of this study. A wearable setup should use a sensor fusion approach with IMUs to only activate the magnetic system when required. A similar power management scheme was used with UWB sensors [23].

In summary, there are two applicable strategies for continuing this work. First, the presented minimal setup should be applied to a specific clinical research question, such as the differentiation between PD patients and and healthy controls based on the step width and step width variability. Secondly, additional hardware (sensor placement) and software (calibration, analysis) efforts should be made to enable comprehensive gait kinematics tracking throughout the whole gait cycle.

## 5. Conclusions

In this pilot study, we demonstrated accurate step width estimation with a body-worn magnetoelectric sensor system. Based on considerations regarding step width definitions, we adapted the system and implemented a geometric approach to extract the relevant step times and widths from the magnetic distance estimates. In a direct comparison with device-mounted OMC markers during the swing phase (shank clearance), the system achieved a high accuracy and precision. In a more general comparison with heel markers during the swing phase, the system still maintained high precision and correlation for the individual feet, but the accuracy was limited by a proxy bias of a few centimeters, which should be further investigated. The system seems suitable for providing distance estimates as an additional spatial dimension in a sensor fusion approach with IMUs. Our results highlight the potential of this magnetic approach for gait stability assessment in clinical and home assessment conditions.

## Figures and Tables

**Table 1:** Overview of study demographics and parameters. Values stated as mean with one standard deviation. Averages obtained from the initial contact reference.

Category	Metric	Value
Participants	Number	8
	Age	27.5 ± 2.4 years
	Sex	3 (f), 5 (m)
Walking conditions	Speed	0.5 m/s
	Duration	120 s
Results (average)	Number of steps	146 ± 13
	Step width	9.76 ± 3.22 cm
	Step width variability	1.26 ± 0.26 cm

**Table 2:** Distance estimation performance of the magnetic motion tracking system compared to the rigid body of optical markers directly attached to each device. The devices are actuator 0 (A0, right shank), sensor 0 (S0, left lower shank) and sensor 1 (S1, left upper shank). Metrics are provided as averages across participants. Bias: Mean of the (signed) error. SD: Standard deviation of the (signed) error. MAE: Mean absolute error. RMSE: Root mean squared error. SCC: Spearman correlation coefficient.

Distance	Bias (cm)	SD (cm)	MAE (cm)	RMSE (cm)	SCC
Lower distance (A0-S0)	−0.5	0.30	0.5	0.6	1.00
Upper distance (A0-S1)	−0.1	0.30	0.3	0.4	0.98
All distances	−0.3	0.37	0.4	0.5	0.99

**Table 3:** Temporal performance of the magnetic estimate compared to the shank clearance and mid-swing reference methods. Metrics are provided as averages across participants. Bias: Mean of the (signed) error. SD: Standard deviation of the (signed) error. MAE: Mean absolute error. RMSE: Root mean squared error.

Detected Time Points	Bias (ms)	SD (ms)	MAE (ms)	RMSE (ms)
Compared to shank clearance reference				
Lower distance minima (A0-S0)	6	8	7	10
Upper distance minima (A0-S1)	0	13	10	14
All minima	3	11	9	12
Compared to mid-swing reference				
Lower distance minima (A0-S0)	3	13	11	14
Upper distance minima (A0-S1)	6	21	19	23
All minima	5	18	15	19

**Table 4:** Step width estimation performance of the magnetic motion tracking system. Metrics are provided as averages across participants. Bias: Mean of the (signed) error. SD: Standard deviation of the (signed) error. MAE: Mean absolute error. RMSE: Root mean squared error. SCC: Spearman correlation coefficient. MAE-VAR: MAE of the step width variability.

Steps	Bias (cm)	SD (cm)	MAE (cm)	RMSE (cm)	SCC	MAE-VAR (cm)
Compared to shank clearance reference						
Left	−1.0	0.35	1.0	1.2	0.92	0.09
Right	0.1	0.23	0.2	0.3	0.95	0.05
All	−0.5	0.68	0.6	0.9	0.93	0.41
Compared to mid-swing reference						
Left	−3.7	0.39	3.7	4.0	0.90	0.12
Right	−4.6	0.47	4.6	4.7	0.88	0.18
All	−4.2	0.75	4.2	4.4	0.82	0.33
Compared to initial contact reference						
Left	−3.7	1.16	3.7	4.2	0.45	0.39
Right	−5.3	1.17	5.3	5.7	0.42	0.37
All	−4.5	1.52	4.5	5.0	0.33	0.25

## Data Availability

All motion data collected in this study are publicly accessible in the Zenodo repository ‘Magnetic Distance Estimation with Magnetoelectric Sensors’ [51]. The data are structured in the Motion-BIDS format for reproducible research [52,53].

## Abbreviations

The following abbreviations are used in this manuscript:

BIDS	Brain imaging data structure
IMU	Inertial measurement unit
KiRAT	Kiel real-time application toolkit
MAE	Mean absolute error
ME	Mean error
ME sensor	Magnetoelectric sensor
MEMS	Micro-electromechanical system
OMC	Optical motion capture
PD	Parkinson’s disease
(R)MSE	(Root) mean squared error
SCC	Spearman correlation coefficient
SD	Standard deviation
SNR	Signal-to-noise ratio
UWB	Ultra wideband
